# Transnasal humidified rapid-insufflation ventilator exchange compared with laryngeal mask airway for endoscopic thoracic sympathectomy: a randomized controlled trial

**DOI:** 10.3389/fmed.2023.1252586

**Published:** 2023-12-05

**Authors:** Chunmei Lin, Dandan Wang, Yulu Yan, Ruihan Zhong, Chaoyang Li, Jie Zhang

**Affiliations:** Department of Anesthesiology, Huazhong University of Science and Technology Union Shenzhen Hospital, Shenzhen, China

**Keywords:** transnasal humidified rapid-insufflation ventilator exchange, laryngeal mask airway, endoscopic thoracic sympathectomy, carbon dioxide accumulation, apneic oxygenation

## Abstract

**Background:**

Transnasal humidified rapid-insufflation ventilator exchange (THRIVE) has the characteristics of operating easily and maintaining oxygenation and eliminating CO_2_, which makes it possible to be used in endoscopic thoracic sympathectomy (ETS). The application of THRIVE in ETS remains undefined. The purpose of this randomized controlled study is to assess the efficacy between THRIVE and laryngeal mask airway (LMA) for ETS.

**Methods:**

In total, 34 patients from May 2022 to May 2023 in Huazhong University of Science and Technology Union Shenzhen Hospital undergoing ETS were randomly divided into a THRIVE group (*n* = 17) and an LMA group (*n* = 17). A serial arterial blood gas analysis was conducted during the perioperative period. The primary outcome was the arterial partial pressure of carbon dioxide (PaCO_2_) during the perioperative period. The secondary outcome was arterial partial pressure of oxygen (PaO_2_) during the perioperative period.

**Results:**

The mean (SD) highest PaCO_2_ in the THRIVE group and LMA group were 99.0 (9.0) mmHg and 51.7 (5.2) mmHg, respectively (*p* < 0.001). The median (inter-quartile range) time to PaCO_2_ ≥ 60 mmHg in the THRIVE group was 26.0 min (23.2–28.8). The mean (SD) PaO_2_ was 268.8 (89.0) mmHg in the THRIVE group and 209.8 (55.8) mmHg in the LMA group during surgery (*p* = 0.027).

**Conclusion:**

CO_2_ accumulation in the THRIVE group was higher than that of the LMA group during ETS, but THRIVE exhibited greater oxygenation capability compared to LMA. We preliminarily testified that THRIVE would be a feasible non-intubated ventilation technique during ETS under monitoring PaCO_2._

## 1 Introduction

Hyperhidrosis (HH) is a major dermatologic disease characteristic of producing excessive sweat, occurring in 0.6–1% of the general public and resulting in a negative effect on the patient’s life (1). The best treatment for patients with primary HH is endoscopic thoracic sympathectomy (ETS), especially for the HH of palmar manifestations (2). Endotracheal intubation has been performed to offer patients adequate oxygen during ETS. Hsieh et al. (3) compared 17 patients receiving anesthesia with double-lumen endobronchial tube ventilation and 19 patients receiving anesthesia with laryngeal mask airway (LMA) during ETS for palmar HH, and they found there was no difference in oxygen saturation between the two groups during operation, which makes it possible for LMA to be used in ETS. There are many complications with the use of endotracheal intubation and LMA. Takahata et al. (4) have found a number of problems and complications with airway management by endotracheal intubation and laryngeal mask airway, including hoarseness and arytenoids dislocation by using endotracheal intubation and aspiration, oropharyngeal leak and gastric distension by using LMA. In addition, Bhavani-Shankar et al. (5) described negative pressure injury occurring with the use of LMA during the resection of a ganglion cyst.

Transnasal humidified rapid-insufflation ventilator exchange (THRIVE), a new type of non-intubated ventilation, provides patients with continuous oxygen through a non-invasive high-flow nasal cannula. Compared with traditional apneic oxygenation, carbon dioxide (CO_2_) removal of THRIVE is regulated by the mutual effect of supraglottic flow vortices and flow oscillation created by cardiogenic oscillation (6). The first application of THRIVE was in neonates for the treatment of apnea and the prevention of extubation failure (7). In addition, it is generally considered that THRIVE can be used in intensive care units and the induction of anesthesia to supply oxygen therapy for patients (8, 9). Moreover, several groups have successfully applied THRIVE in patients with microlaryngoscopic surgery (10, 11). In a recent prospective study, we have indicated that THRIVE can be an effective and safe ventilation way for 19 Chinese patients performing microlaryngoscopic surgery (12). Furthermore, Liu et al. (13) have used THRIVE for oxygenation in thoracoscopic segmentectomy and exhibited that it can maintain oxygen reserves during surgery. ETS has the characteristics of short operation time and good treatment effect for primary HH. At present, the main airway management methods in ETS are tracheal intubation (including single-lumen tube and double-lumen tube) and laryngeal mask airway. These airway management methods have damage to the trachea. As a non-tracheal intubation mode of ventilation, THRIVE has no damage to the airways. However, THRIVE can cause carbon dioxide accumulation after prolonged use. We have found that the PaCO_2_ increased by 1.68 ± 0.12 mmHg every minute linearly in non-laser microlaryngoscopic surgery (12). Moreover, thoracoscopic surgery may involve the absorption of CO_2_ into blood, which increases the risk of hypercapnia. Therefore, whether THRIVE can be safely and reliably applied to ETS requires further research.

In this study, we performed a randomized controlled study to evaluate the efficacy between THRIVE and LMA for ETS and tried to provide more evidence about the application of THRIVE in ETS for the Chinese population.

## 2 Materials and methods

### 2.1 Methods

This study was conducted from May 2022 to May 2023 in Huazhong University of Science and Technology Union Shenzhen Hospital. It was approved by the Chinese Clinical Trial Registry (ChiCTR2200061446). The written consent of all participants was required before performing the experiment.

### 2.2 Study object

**Figure 1:**
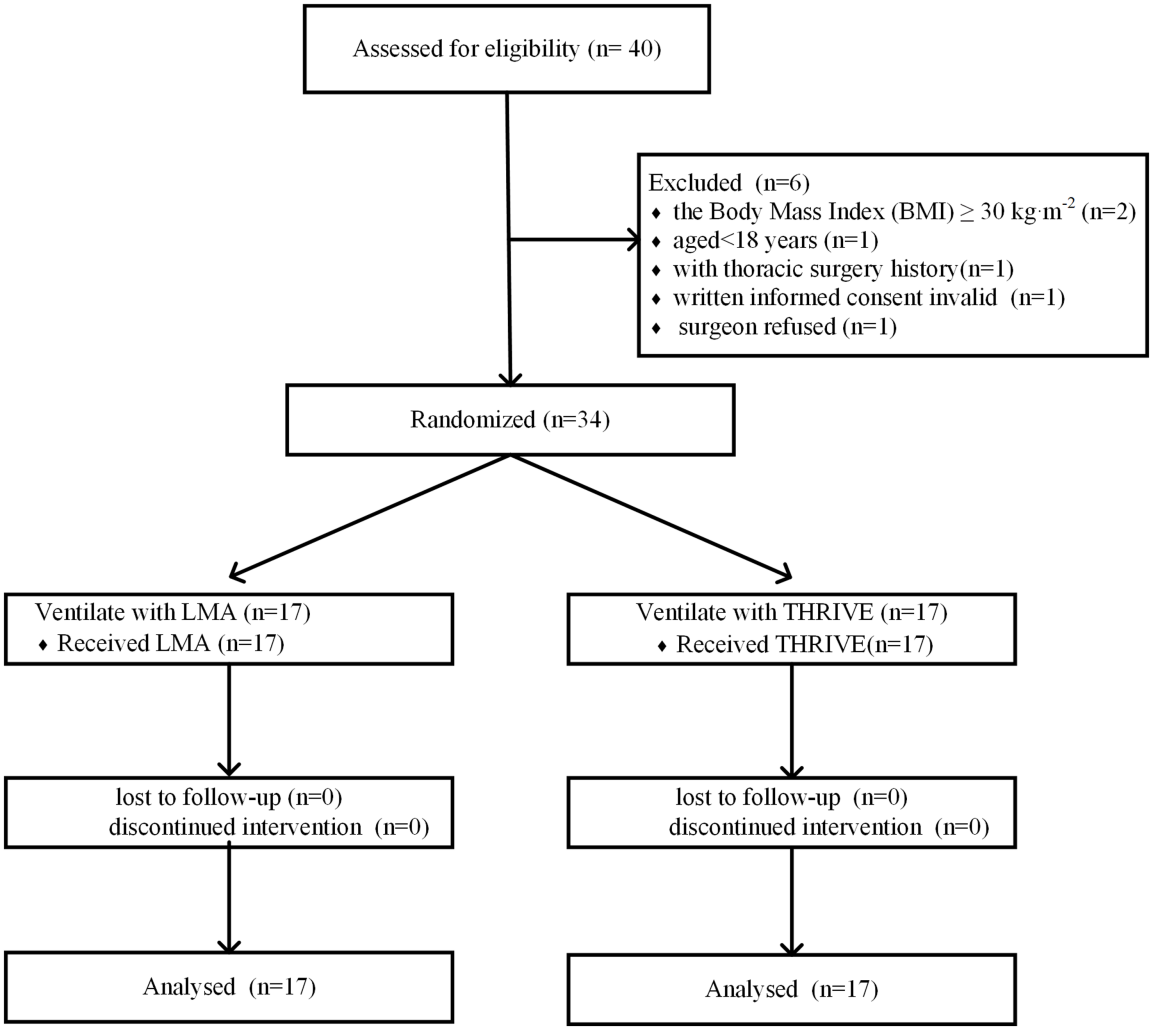
Consolidated standards of reporting trials flow diagram. THRIVE, Transnasal humidified rapid-insufflation ventilator exchange; LMA, Laryngeal mask airway.

After meeting the inclusion criteria, 34 patients were recruited and randomized into the THRIVE group (*n* = 17) and the LMA group (*n* = 17; Figure 1). In total, 34 patients presenting for both sides of uniportal EST were recruited. Patients were randomly assigned to the THRIVE group and LMA group in a 1:1 ratio. Investigators (YY) conducted the block randomization by using a computer and placed the identifier in opaque sealed envelopes. After patients arrived in the operating room with a sealed envelope, the anesthesiologist (JZ or CLin) opened the envelopes and performed the assigned intervention. The inclusion criteria were as follows: primary HH, elective surgery within 1 h, aged 18–60 years, American Society of Anesthesiologists (ASA) physical status 1–2. The exclusion criteria were as follows: the body mass index (BMI) of ≥30 kg*·*m^−2^, a Mallampati grade of 3–4, and the patient with extensive pleural adhesion or pleural hypertrophy or thoracic surgery history.

### 2.3 Study protocol

All patients were monitored for the following indicators before and during surgery, including electrocardiogram, invasive blood pressure, saturation of peripheral oxygen (SpO_2_), bispectral index (BIS), arterial partial pressure of carbon dioxide (PaCO_2_), arterial partial pressure of oxygen (PaO_2_), and potential of hydrogen (pH). In the THRIVE group, the THRIVE was applied to offer patients oxygen with a concentration of 95% at 50 L/min before the administration of anesthetics (Figure not shown). In the LMA group, pre-oxygenation was conducted with a mask at a 5 L/min flow of 100% oxygen. All participants were induced by using propofol, sufentanil, and vecuronium until a BIS value of less than 60. A laryngeal mask airway was established in the LMA group after pre-oxygenation for 3–5 min (Figure not shown). The tidal volume was set at 6–7 mL/kg and the respiratory rate was set at 12–15 breaths/min. In both groups, the maintenance of anesthesia with propofol and remifentanil depended on the BIS value and surgical procedure. Propofol and remifentanil were administered in the target-controlled infusion of the Marsh model. In addition, it is necessary to use parecoxib sodium, tramadol, palonosetron, and dexamethasone as general anesthetic adjuvant drugs. At the beginning of the operation, 1% ropivacaine was used for local anesthesia at the incision. After an incision was made in the fourth intercostal space, the endoscope was inserted into the pleural cavity; thus, we could observe the thoracic sympathetic nerve (Figure not shown). In the THRIVE group, we immediately performed mask ventilation when the SpO_2_ of patients dropped below 95% or the PaCO_2_ accumulated more than 110 mmHg during the procedure. In addition, the elimination of CO_2_ to less than 60 mmHg was carried out by using mask ventilation after the surgery. Meanwhile, THRIVE was discontinued when the consciousness of the patient was regained, and then the patient was transferred to the post-anesthesia care unit (PACU). In the LMA group, the laryngeal mask airway was removed after the recovery of the patient’s consciousness, and then the participant was transported to PACU.

### 2.4 Data collection and analyses

The primary outcome was the measurement of PaCO_2_ during the perioperative period. Secondary outcomes were (1) the measurements of PaO_2_ and pH at seven test points, respectively; (2) time to PaCO_2_ ≥ 60 mmHg; (3) the number of intraoperative ventilations by mask; (4) the number of pharyngalgia after operation. The seven time points were defined as follows: before pre-oxygenation (T_0_), after an induction (T_1_), at the beginning of operation (T_2_), at the end of sympathetic nerve excision on one side (T_3_), at the end of sympathetic nerve excision on the other side (T_4_), at the end of the operation (T_5_), and the moment of consciousness recovery (T_6_). Data analysis was performed by using SPSS25.0. The use of mean standard deviation toward continuous data and the application of number (%) to categorical data was conducted in our study. The *t*-test was applied for continuous variables and a value of *p* < 0.05 was identified as statistically significant. Time to PaCO_2_ ≥ 60 mmHg was conducted by Kaplan–Meier curves and compared between the THRIVE group and the LMA group by the log-rank statistic. Median time to PaCO_2_ ≥ 60 mmHg and 95% confidence intervals (CIs) are shown.

## 3 Results

No differences were observed in the patient characteristics between the groups (Table 1). No differences were observed in PaCO_2_, PaO_2_, and SPO_2_ before pre-oxygenation (Table 2). As shown in Table 3, the mean (SD) highest PaCO_2_ in the THRIVE group was 99.0 (9.0) mmHg, while the mean highest PaCO_2_ was 51.7 (5.2) mmHg in the LMA group, and the difference was statistically significant (*p* < 0.001). The mean (SD) lowest PaO_2_ during surgery in the THRIVE group was 268.8 mmHg (89.0 mmHg), while that of the LMA group was 209.8 mmHg (55.8 mmHg), and the difference was statistically significant (*p* = 0.027). There was no difference in the lowest SpO_2_ during surgery between groups. During the surgery, three patients among 17 participants were performed mask ventilation in the THRIVE group, while none of the patients in the LMA group required intraoperative mask ventilation. In addition, after the operation, two patients in the LMA group had pharyngalgia, while none had pharyngalgia in the THRIVE group.

**Table 1:** Patients and intraoperative characteristics.

	THRIVE (*n* = 17)		LMA (*n* = 17)		*p*
Age, years	26.6	8.7	23.6	4.6	0.219
Weight, kg	57.4	9.8	58.0	10.5	0.854
BMI, kg/m^2^	21.1	2.3	21.3	3.2	0.84
Female, *n* (%)	10 (59)		10 (59)		1
ASA, *n* (%)	10 (59)		9 (53)		0.739
I	10		9		
II	7		8		
Surgery duration, min	18.4	8.8	19.9	6.6	0.571

Values are mean ± standard deviation or *n* (%); BMI, Body mass index; ASA, American society of anesthesiologists; *n* (%), number (0%); THRIVE, Transnasal humidified rapid-insufflation ventilator exchange; and LMA, Laryngeal mask airway.

**Table 2:** Baseline arterial blood gas data and oxygen saturation.

	THRIVE (*n* = 17)		LMA (*n* = 17)		*p*
PaCO_2_ in room air, mmHg	37.7	3.6	38.6	5.0	0.561
PaO_2_ in room air, mmHg	96.4	6.5	100.3	10.5	0.198
SpO_2_ in room air, %	98.5	1.6	98.6	1.0	0.930

Values are mean ± standard deviation; PaCO_2_, Arterial partial pressure of carbon dioxide; PaO_2_, Arterial partial pressure of oxygen, SpO_2_, Saturation of peripheral oxygen; THRIVE, Transnasal humidified rapid-insufflation ventilator exchange; LMA, Laryngeal mask airway.

**Table 3:** Study outcomes.

	THRIVE (*n* = 17)		LMA (*n* = 17)		*p*
Highest PaCO_2_ during operation, mmHg	99.0	9.0	51.7	5.2	0.000
Lowest PaO_2_ during operation, mmHg	268.8	89.0	209.8	55.8	0.027
Lowest SpO_2_ during operation, %	99.1	1.4	99.6	0.8	0.139
Number of intraoperative mask ventilation, *n* (%)	3 (20)		0 (0)		0.073
Number of pharyngalgia after operation, *n* (%)	0 (0)		2 (12)		0.154

Values are mean ± standard deviation or *n* (%). PaCO_2_, Arterial partial pressure of carbon dioxide; SpO_2_, Saturation of peripheral oxygen; PaO_2_, Arterial partial pressure of oxygen; THRIVE, Transnasal humidified rapid-insufflation ventilator exchange; LMA, Laryngeal mask airway.

**Figure 2:**
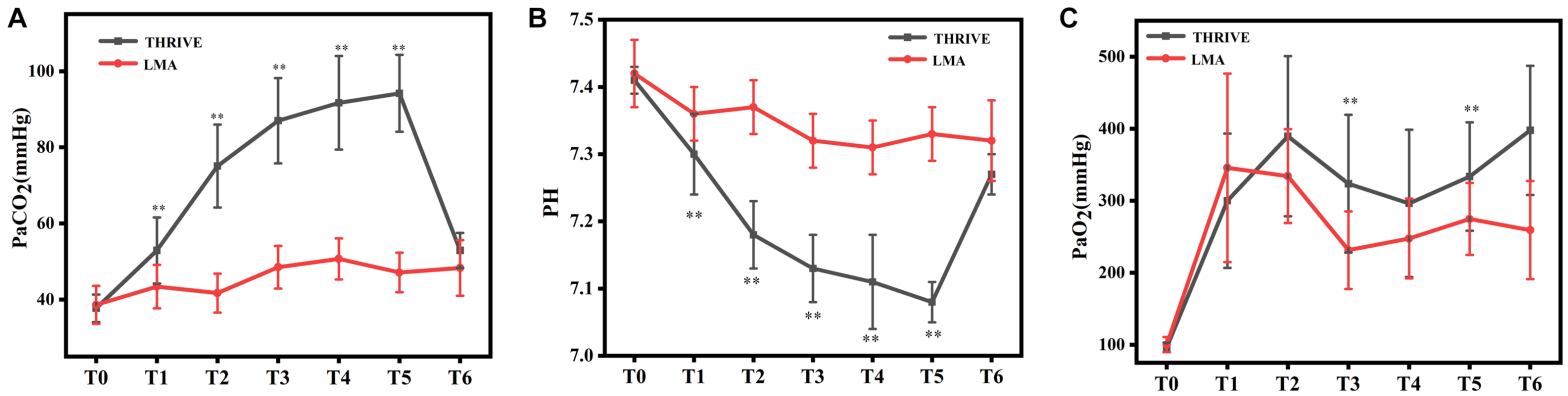
Arterial blood gas analysis. PaCO_2_ **(A)**, pH **(B)**, and PaO_2_ **(C)** in seven test points between THRIVE and LMA groups during the perioperative period. T_0_: before pre-oxygenation, T_1_: after an induction, T_2_: at the beginning of the operation, T_3_: at the end of sympathetic nerve excision on one side, T_4_: at the end of sympathetic nerve excision on the other side, T_5_: at the end of the operation, and T_6_: the moment of consciousness recovery. THRIVE, Transnasal humidified rapid-insufflation ventilator exchange; LMA, Laryngeal mask airway; PaCO_2_, Arterial partial pressure of carbon dioxide; pH, The potential of hydrogen; and PaO_2_, Arterial partial pressure of oxygen. ^**^p < 0.01 vs. LMA group.

The tendency of PaCO_2_ during the perioperative period is presented in Figure 2A. PaCO_2_ in the THRIVE group increased by 56.5 mmHg from T_0_ to T_5_ and dropped below 60 mmHg at T6. PaCO_2_ in the LMA group showed no significant change during the perioperative period. A statistically relevant difference in PaCO_2_ was observed between the two groups during operation (*p* < 0.01). As shown in Figure 2B, pH in the THRIVE group decreased from T_0_ to T_5_ and rose to approximately 7.3 at T_6_. There was no significant fluctuation in the pH of LMA patients, and a statistically relevant difference was observed between the two groups during surgery (*p* < 0.01). As shown in Figure 2C, the PaO_2_ of both groups increased significantly after pre-oxygenation. Moreover, PaO_2_ of the THRIVE group achieved higher levels compared to the LMA group during operation, and a significant difference was observed between the two groups at T_3_ and T_5_ (*p* < 0.01).

**Figure 3:**
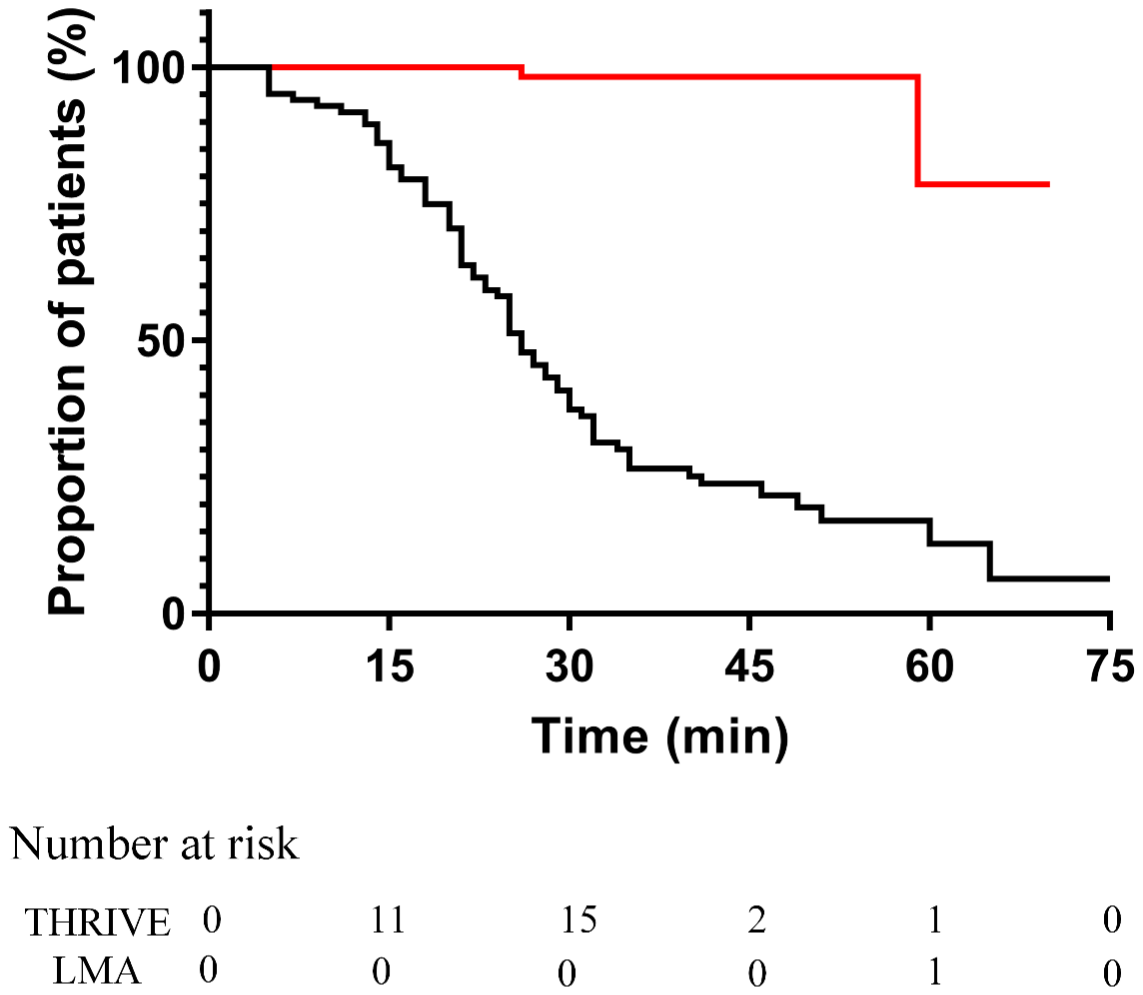
Kaplan–Meier curves of time to PaCO_2_ ≥ 60 mmHg in the THRIVE group (black line) and LMA group (red line). The hazard ratio comparing between THRIVE and LMA groups was 41.67 (95% confidence interval, 10.31–166.7; Log-rank *p* < 0.01). THRIVE, Transnasal humidified rapid-insufflation ventilator exchange; LMA, Laryngeal mask airway.

As shown in Figure 3, the Median (inter-quartile range) time to PaCO_2_ ≥ 60 mmHg in the THRIVE group was 26.0 min (23.2–28.8). The risk of PaCO_2_ ≥ 60 mmHg was significantly higher in the THRIVE group than in the LMA group (hazard ratio = 41.67; 95% CI 10.31–166.7; Log-rank *p* < 0.01).

## 4 Discussion

Transnasal humidified rapid-insufflation ventilator exchange can remove CO_2_ through by the mutual effect of supraglottic flow vortices and flow oscillation created by cardiogenic oscillation (6). Recent studies (9, 14) have proved the lower accumulation rates of CO_2_ with THRIVE compared with historical (15), which has implied that THRIVE can produce the clearance of CO_2_. However, the CO_2_ created by anesthetic techniques in historical studies and the analysis method of recent studies have made the results contentious. In our study, we have found that the mean highest concentration of THRIVE group was 99.0 mmHg. This verified that the major limiting factor of using THRIVE in ETS is hypercarbia. According to the Henderson–Hasselbalch equation, the degree to which respiratory acidosis affects the body is significantly associated with hypercapnia. Although CO_2_ drops quickly postoperatively, the serious consequence of short-term respiratory acidosis requires vigilance, especially for gerontic patients with cardiovascular disease. During the perioperative period, severe hypercapnia can result in clinical adverse events (16–18). Kerr et al. (16) reported an unusual case of hypercapnia and surgical emphysema during transanal endoscopic microsurgery, which led to delayed postoperative ventilatory failure. A study done by Son et al. (17) found that intraoperative hypercarbia may be related to a high incidence of postoperative nausea and vomiting (PONV). In addition, Kim et al. (18) indicated that the circulatory response to hypercapnia is an increase in arterial pressure and heart rate. Therefore, the application of THRIVE in ETS should be limited to young patients without serious cardiovascular and cerebrovascular diseases. It is beneficial to use a transcutaneous CO_2_ monitor during operation.

The duration of apnea will extend with THRIVE by eliminating CO_2_, but several studies have indicated that CO_2_ will accumulate as the use of THRIVE prolongs (12, 19). Booth et al. (19) have shown that CO_2_ accumulation in the apnea group was double than that of the spontaneous ventilation group after 30 min with the application of THRIVE. In addition, Ma et al. (12) have found that CO_2_ increased to 112 mmHg when the apnea time extended to 65 min. The level of PaCO_2_ less than 60 mmHg has been reported without serious adverse reaction (12). In our study, we found that the median time to PaCO_2_ ≥ 60 mmHg in the THRIVE group was 26.0 min. Moreover, thoracoscopic surgery may involve the absorption of CO_2_ into blood, which increases the risk of hypercapnia. Therefore, the duration of operation should be strictly controlled to avoid severe hypercapnia. Hypoxia is more likely to occur in obese patients due to decreased functional residual capacity and lung compliance as well as increased oxygen consumption and respiratory work. Additionally, 10% of obese patients have difficulty with mask ventilation because of anatomic factors. Although Wu et al. (20) have indicated that PaO_2_ achieved a higher level with the use of THRIVE in obese patients, one study showed that high BMI patients were prone to hypoxemia (21). Therefore, only patients with a BMI less than 30 were included in our study to ensure safety.

HH is a disease that can negatively affect patients’ quality of life, and some studies have shown that the best treatment for patients with primary HH is endoscopic thoracic sympathectomy. The main airway management methods in ETS are tracheal intubation (including single-lumen tube and double-lumen tube) and laryngeal mask airway. There is currently no research on the application of THRIVE to ETS. LMA has been used in thoracic surgery due to its good oxygenation capability and convenient airway management (5); however, the adverse effects created by LMA are not trivial. THRIVE can deliver humidified air to the patients at 37°C and 100% humidity through nasal ducts. Several studies have verified that THRIVE can prevent the reduction of intraoperative temperature and maintain oxygen reserve during thoracoscopic surgery for patients with early lung cancer (22, 23). This is the first study to evaluate the effect of THRIVE and LMA for ETS. Compared to LMA, THRIVE can lead to a higher CO_2_ accumulation but has greater oxygenation capability during ETS. Our study preliminarily testified that THRIVE can be availably utilized in ETS under CO_2_ monitoring.

As shown in Figure 2, PaCO_2_ increased from 37.7 mmHg at T_0_ to 94.2 mmHg at T_5_ in the THRIVE group, while PaCO_2_ did not change significantly in the LAM group. These results indicate that we should be alert to the occurrence of hypercapnia when applying THRIVE for ETS. PaCO_2_ should be closely monitored with the use of THRIVE, and if necessary, mask ventilation should be used to promote the emission of carbon dioxide. Additionally, PaCO_2_ in the THRIVE group decreased rapidly to below 60 mmHg after the surgery, indicating that THRIVE can be safely used in ETS for young patients with BML < 30 Kg/m^2^. The contraction and relaxation of the lungs can affect the exposure of the surgical field with the application of LMA during ETS, and the anesthesiologist may be required to temporarily suspend artificial ventilation of the patient to minimize the impact of lung movement on the surgical exposure. However, this problem does not exist when choosing THRIVE. In our study, we found that two out of 17 patients in the LAM group experienced postoperative pharyngalgia, which may be caused by a laryngeal mask to the pharyngeal tissue. This adverse reaction will affect postoperative patient comfort. However, no postoperative pharyngalgia was observed in the THRIVE group.

Transnasal humidified rapid-insufflation ventilator exchange can transport a high concentration of oxygen by combining a high fraction and high flow of inspired oxygen and produce positive pharyngeal pressure to enhance oxygenation (24). Several studies have reported that THRIVE can effectively prevent the happen of arterial desaturation (21, 25). In our study, the PaO_2_ of both groups increased significantly after pre-oxygenation, and the PaO_2_ of the THRIVE group achieved higher levels compared to the LMA group during operation, which proved the powerful ability of THRIVE to improve oxygenation. Our study showed that SpO_2_ of THRIVE group was greater than 95% during the perioperative period (data not shown). However, Vourc’h et al. (26) manifested that THRIVE promoted the occurrence of desaturation below 95% by generating a lower concentration of end-tidal oxygen. These discrepancies might be due to the differences in the measurement of desaturation and the oxygenation protocol. In addition, the progressive decrease of PaO_2_ during operation and the gradual rise of PaO_2_ with the end of surgery were observed in our study. Intrapulmonary shunt and pulmonary atelectasis may be the possible reason, which is a common phenomenon with the administration of anesthetics (24). The delivery of high oxygen concentration by THRIVE may also result in atelectasis (27). Moreover, artificial pneumothorax caused by surgery can lead to atelectasis and PaO_2_ reduction.

We should pay more attention to the following limitations of our study. First, only 34 patients were enrolled in our study, and it is important to include more data to verify the feasibility of THRIVE in ETS. Second, there is no double blindness between the anesthesiologist and the data analyst, which may affect the objectivity of the results. Third, the mean age of the patients included in our study was young adults and BMI was less than 30 kg/m^2^, and the availability of THRIVE in ETS for older patients with BMI greater than 30 kg/m^2^ remains uncertain. Fourth, the feeling of the surgeon during the operation (mainly refers to the impact of breathing on the exposure of the surgical field) will be an important factor in evaluating the effectiveness of using THRIVE, which should be noticed in future. Finally, real-time monitoring of exhaled CO_2_ could not be done increasing the risk to patients. Moreover, the applicability of findings to other case scenario may be different.

## 5 Conclusion

We have conducted a randomized controlled study that THRIVE would be an effective and safe non-intubated ventilation technique during ETS under monitoring PaCO_2._ Although THRIVE exhibited significantly greater CO_2_ accumulation compared to LMA during operation, THRIVE presented better apneic oxygenation capability than LMA. These findings indicate that THRIVE may be regarded as an alternative anesthetized technique for young and BMI less than 30 Kg/m^2^ patients undergoing ETS. Further studies are needed to evaluate the efficacy of THRIVE for older patients with a BMI greater than 30 Kg/m^2^. This will help to determine the appropriate population for THRIVE during ETS and improve clinical safety.

## Data availability statement

The original contributions presented in the study are included in the article/supplementary material, further inquiries can be directed to the corresponding authors.

## Ethics statement

The studies involving humans were approved by the Chinese Clinical Trial Registry. The studies were conducted in accordance with the local legislation and institutional requirements. The participants provided their written informed consent to participate in this study. Written informed consent was obtained from the individual(s) for the publication of any potentially identifiable images or data included in this article.

## Author contributions

CLin, DW, and JZ: study design. CLin, DW, YY, and RZ: information collection. CLin and DW: analysis of data. CLin: manuscript drafting. CLin, DW, YY, RZ, CLi, and JZ: manuscript revision, editing, and approval. All authors contributed to the article and approved the submitted version.
